# Zein-Based Nanoparticles as Active Platforms for Sustainable Applications: Recent Advances and Perspectives

**DOI:** 10.3390/nano14050414

**Published:** 2024-02-23

**Authors:** Emilia Oleandro, Mariamelia Stanzione, Giovanna Giuliana Buonocore, Marino Lavorgna

**Affiliations:** 1 Institute of Polymers, Composites and Biomaterials-CNR, Piazzale E. Fermi 1, 80055 Portici, Italy; emilia.oleandro@ipcb.cnr.it (E.O.); giovannagiuliana.buonocore@cnr.it (G.G.B.); marino.lavorgna@cnr.it (M.L.); 2 Institute of Polymers, Composites and Biomaterials-CNR, Via Previati 1/E, 23900 Lecco, Italy

**Keywords:** nanotechnology, nanoparticles, protein-based carriers, zein, sustainability

## Abstract

Nanomaterials, due to their unique structural and functional features, are widely investigated for potential applications in a wide range of industrial sectors. In this context, protein-based nanoparticles, given proteins’ abundance, non-toxicity, and stability, offer a promising and sustainable methodology for encapsulation and protection, and can be used in engineered nanocarriers that are capable of releasing active compounds on demand. Zein is a plant-based protein extracted from corn, and it is biocompatible, biodegradable, and amphiphilic. Several approaches and technologies are currently involved in zein-based nanoparticle preparation, such as antisolvent precipitation, spray drying, supercritical processes, coacervation, and emulsion procedures. Thanks to their peculiar characteristics, zein-based nanoparticles are widely used as nanocarriers of active compounds in targeted application fields such as drug delivery, bioimaging, or soft tissue engineering, as reported by others. The main goal of this review is to investigate the use of zein-based nanocarriers for different advanced applications including food/food packaging, cosmetics, and agriculture, which are attracting researchers’ efforts, and to exploit the future potential development of zein NPs in the field of cultural heritage, which is still relatively unexplored. Moreover, the presented overview focuses on several preparation methods (i.e., antisolvent processes, spry drying), correlating the different analyzed methodologies to NPs’ structural and functional properties and their capability to act as carriers of bioactive compounds, both to preserve their activity and to tune their release in specific working conditions.

## 1. Introduction

Innovative approaches to nanotechnology involve the development and manipulation of nanometer-sized materials characterized by high surface areas, improved adsorption capacities, and biochemical reactivities. These features make nanotechnology approaches suitable for designing nanocarriers for incorporation, tailored delivery, and the specific targeting of active agents. Such nanoscale systems are capable of protecting the active agent from rapid degradation and exposure to atmospheric agents, prolonging its release from the material in which it is embedded, i.e., a composite coating, to the target substrate, and enhancing its efficacy. Currently, nanocarriers are being developed to make active systems to release suitable compounds that can counteract specific targeting mechanisms such as oxidation, corrosion, the proliferation of cancer cells, and issues that commonly occur in specific sectors such as food storage, resulting in the protection of metal substrates and innovative anticancer therapies [1,2,3]. Nanocarriers are classified according to their composition in inorganic-, organic-, and carbon-based nanoparticles [4]. Inorganic particles are produced from pure metals, metal oxides, semiconductors, and ceramics, while organic particles are obtained from proteins, lipids, polymers, carbohydrates, or other organic compounds, and are divided into synthetic or natural groups. The choice of an appropriate material for nanoparticle development depends on factors such as particle size, interaction with the active compounds, desired release profile, and surface charge [5].

Within this context, the use of sustainable polymers from both renewable sources and food byproducts can be beneficial for potentially reducing the pollution generated by synthetic materials. Among different biomaterials, proteins can be considered to be the most promising alternatives to conventional petrol-based materials, not only for their non-toxicity and high biocompatibility, but mainly due to both the fine tailoring of particle size distribution, with smaller particle sizes compared to those obtained with other materials [2,6,7,8,9,10], and their enhanced affinity to bioactive compounds via a variety of molecular interactions, such as hydrogen bonds, hydrophobic and π-π interactions, and disulfide bonds [11]. Bio-based nanoparticles can be utilized as nanocarriers by encapsulating active compounds for potential applications in several sectors, including the medical, pharmaceutical, food packaging, and agricultural fields [12,13].

Protein-based nanoparticles are typically derived from animal or plant proteins, with vegetable proteins gaining increasing popularity due to their environmentally friendly features which prevent further human and animal issues. Within this frame, in order to encapsulate, preserve, and deliver bioactive compounds, a significant academic and industrial interest is growing toward the use of proteins such as zein, gliadin, soy, glutenin, and lectin, which are extracted from corn, barley, soybean, wheat flour, and germ, respectively, and include sunflower, pea, and potato seed proteins [14].

**Figure 1:**
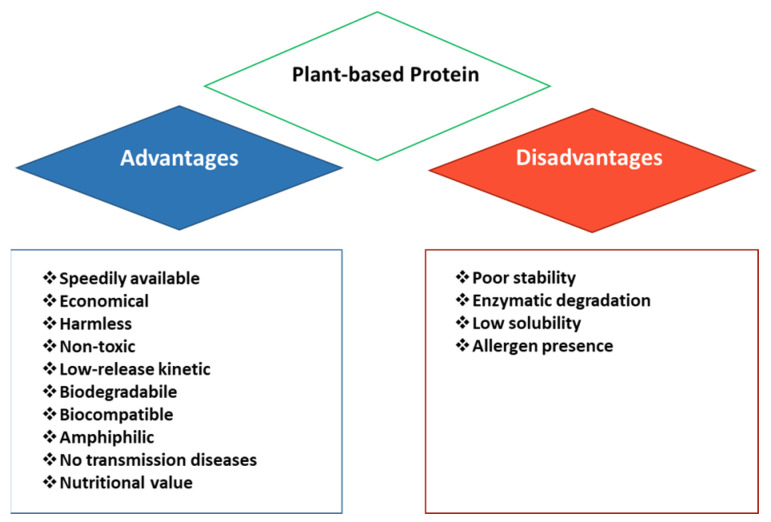
Advantages and disadvantages of plant proteins.

Unlike animal proteins, plant proteins have multiple advantages, including the ability to provide sustained release of the active compound due to their hydrophobic nature, the ability to be produced without the use of chemical and/or physical treatments or chemical bonding molecules, and their availability in large quantities at an affordable price (Figure 1). Protein-based carriers also have the advantage both of enabling the encapsulation of both hydrophilic and hydrophobic active compounds and providing added nutritional value when used as a component of fertilizers or insecticides for targeting applications in the agriculture sector [15,16,17,18]. However, the use of protein-based nanoparticles also has several limitations, such as poor stability, enzymatic degradation, low solubility, and the presence of allergens, particularly during oral administration to counter specific diseases [7,19,20].

Among plant-based proteins, zein is characterized by a marked amphiphilic character due to the balance between hydrophobic and hydrophilic groups within its structure. Its solubility in hydroalcoholic solutions induces a very interesting effect on structural reconfiguration at the nanometer scale as a function of the specific solvent, as well as solvent removal. A further advantage associated with zein nanoscale configuration is represented by the presence of positive charges that are capable of encapsulating negatively charged active compounds; furthermore, its wide range of isoelectric points represents a suitable chemical trigger for the delivery of different drugs, foods, and nutrients [21].

The main scope of the present review is to provide a comprehensive overview of both the technologies implemented for the production of zein-based nanoparticles and their recent applications in agriculture, food/food packaging, and cosmetics, considered as the most front-end industrial fields, excluding the health sector, for which the protein-based nanocarriers have been easily developed and validated. This review confirms that zein-based nanocarriers, as a replacement for synthetic polymers that are undoubtedly harmful to both humans and the environment, can be designed and engineered as promising platforms where it is possible to control the size, interfacial interaction with the surrounding environment, loading capacity, and release kinetics of active compounds to create smart materials.

## 2. Zein: Structure, Properties, and Current Applications

**Figure 2:**
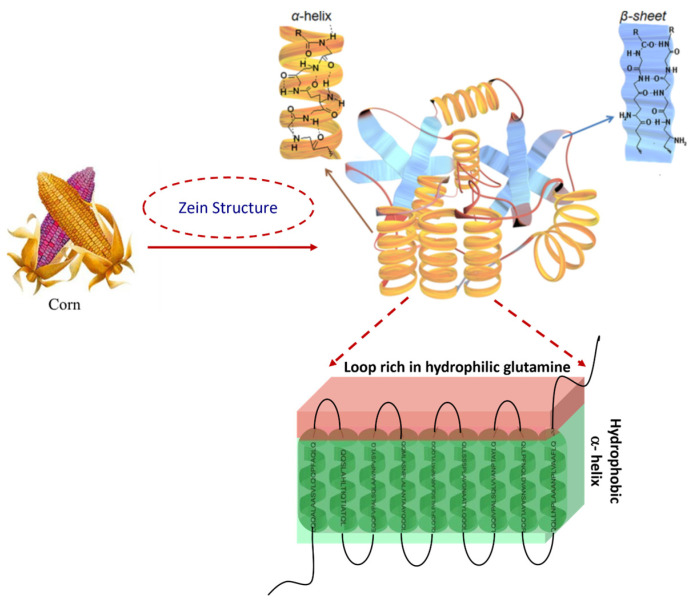
Three-dimensional structural representation of zein, adapted from [31].

Zein (ZN) is a prolamin protein located in the corn endosperm and extracted during maize processing (Figure 2). Zein is characterized by unique and specific properties related to the high presence (50%) of non-polar hydrophobic amino acid residues, such as glutamine, leucine (Leu), proline, and alanine [22]. Based on its solubility properties in hydroalcoholic solution and conformational arrangement, zein can be divided into four main types, classified as α, β, γ, and δ [23]. The α-ZN domain (75–85% of the total fraction) is the dominant peptide with a molecular mass of approximately 19 and 22 kDa (Z19 and Z22, respectively) [24], comprising alcohol-soluble dimers and oligomers and a repeating-unit amino acid sequence. This kind of zein conformation exhibits the highest hydrophobic properties [25]. The different amino acid sequences determine different solubility behaviors and the presence of polar and non-polar amino acids makes it soluble in limited solvents; therefore, zein is not water-soluble, but it is soluble in alkaline solutions (pH ≥ 11.5) [26] and in 70–95% aqueous ethanol solutions [27]. Its properties depend on both the composition of the amino acids and the molecular structures at the nanoscale which is approximately 50–60% of the α-helical form [28]. In particular, the structure of α-zein appears to be similar to an elongated rod, consisting of 10–11 α-helical segments folded upon themselves and connected by hydrogen bonds and glutamine turns [29]. γ-ZN, the second most abundant zein (10–20%), contains a high cysteine content and a possible arrangement of repeating hexapeptide close to the N terminus; instead, both the β- (14 kDa) and δ-ZN (10 kDa) lack repeating-sequence motifs [30].

Academic and industrial interest has been developing in relation to the use of zein as a polymeric material, as it shows several beneficial properties in different fields of application, such as biodegradability and sustainability [32], as well as hydrophobicity, insolubility, and barrier properties to moisture and solutes [33].

Taking advantage of specific properties, zein is widely used in the production of coatings, films, packaging, and particles compared to other cereal proteins such as polysaccharides and lipids [34]. For example, due to its good barrier and hydrophobic properties, zein can be considered as a promising material for biodegradable and eco-friendly packaging [35], playing an important role in the production of food coatings, both to protect their freshness and to preserve their organoleptic properties. In fact, coatings of fruit and vegetables with a layer of plasticized zein delays processes such as germination, ripening, and rancidity, reducing moisture loss and inhibiting microbial growth, while improving their shelf life [36]. Recently, zein has been used in the cultural heritage sector to develop a sustainable spray coating for the protection of Pietra Serena substrates. The results show that the zein film leads to a good hydrophobicity, reaching WCAs around 120°, and to a slight decrease in water vapor permeability; the stone retains its ability to permeate natural vapors [37].

However, although its hydrophobic properties make zein protein a good material for film formation [38], characterized by high transparency, an oxygen barrier, and humidity resistance, the use of zein as a polymetric matrix could suffer from fragility, easy breakage, and low flexibility, which limit its use [39]. Therefore, the addition of additives such as plasticizers [40,41], nanoparticles [42,43], polyphenols [44,45], and biopolymers is used to improve its properties [46].

In addition to the production of biodegradable films or coatings, zein is also used to protect protein-based nanocarrier systems of poorly water-soluble active ingredients, improving their solubility and bioavailability [47], while readily inducing self-assembling into micro/nanoparticles [26,48,49].

Currently, zein-based nanocarriers are used for the encapsulation of both hydrophobic and hydrophilic active compounds. As the zein surface charge is a function of the pH, properly designed pH changes can promote electrostatic interactions with hydrophilic molecules with opposite surface charge, resulting in improved encapsulation efficiency (EE) [50].

In particular, variation in pH can influence the development of zein NPs and their properties, thus affecting the performance of the final product. Podaralla et al. investigated the effect of the pH in the aqueous phase on the precipitation kinetics of zein nanoparticles loaded with 6,7-dihydroxycoumarin (DHC) as a model hydrophobic compound. It has been shown that the pH can have an effect on the dimensions of the nanoparticles; in particular, for pH values close to the zein isoelectric point (pI), a minimum average diameter is obtained; in contrast, it increases for lower and higher pH values (2 and 12, respectively). It is reasonable to assume that, at different pHs, the structural changes are due to the amino acids and their charge in the protein. Indeed, the formation of aggregates is obtained at pH < pI values; in contrast, at pH > pI, the monomeric form is present. Furthermore, the relative rate of precipitation and the consequent solvent evaporation into the aqueous phase can influence the particle size. The variation in pH also affects the zeta potential and, in particular, shows a positive and a negative charge for pH values below and above 5, respectively [51]. In other words, zein nanoparticles possess a pH-dependent surface charge; therefore, at low pH values, a protonation of the amine and carboxyl groups is promoted, resulting in cationic particles with a high positive zeta potential. Conversely, high pH values lead to pH deprotonation towards a highly negative zeta potential value. The presence of mono- and multivalent ions in aqueous suspensions also affects colloidal stability. 

The presence of monovalent salts promotes the particle aggregation in the case of positive surface charge; in contrast, hydrophobic surface behavior is induced with negative surface charge. In the case of multivalent ions, aggregation occurs for nanoparticles with opposite charges. However, it has been observed that their strong adsorption has led to a charge reversal, with a subsequent new stabilization of the suspensions [52].

It has been demonstrated that ZNPs are not stable at high temperatures, and high ionic strength also affects the encapsulation efficiency of bioactive substances. To overcome this criticality, it has been observed that the use of a core shell as a natural polyelectrolyte, such as hyaluronic acid, pectin, carboxymethyl chitosan, carboxymethyl dextrin, soy polysaccharide, or rubber, stabilizes the nanoparticles; this allows for a good stability of pH and ionic strength, increasing the encapsulation efficiency compared to single ZNPs. Moreover, smaller nanoparticle size leads to a longer release time for the active ingredients [53].

Generally, in the case of essential oils (Eos), which are currently the most interesting sustainable active compounds [26], the encapsulation efficiency could reach about 80–90% due to the strong interaction between zein and chemical structures of essential oil [54].

## 3. Zein-Based Nanocarrier Production

As mentioned above, due to their tertiary structure, zein molecules can be found in aqueous ethanol solutions both as nanoaggregates and in the more conventional configuration. Because of this feature, zein nanoparticles can be easily prepared either by liquid–liquid dispersion or by solvent-induced self-assembly evaporation. Additional methods, implemented to attain nanoparticles, include nanoprecipitation, coacervation, and emulsification [17].

The following is an overview of the most common approaches in the production of zein-based nanoparticles. Notably, this review does not provide an exhaustive list of the available methodologies; rather, it focuses on those techniques that are most thoroughly investigated.

### 3.1. Liquid Antisolvent Process

**Figure 3:**
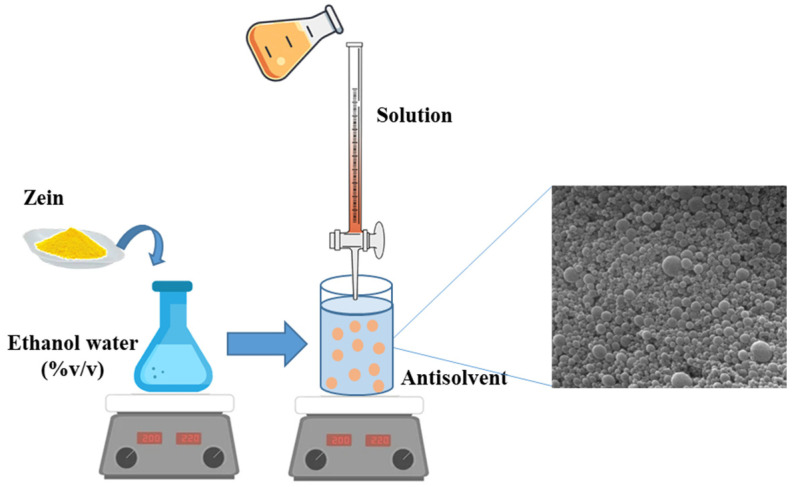
Schematic representation of the liquid antisolvent process and SEM image of zein NPs.

The liquid antisolvent process, also known as liquid–liquid dispersion or phase separation, consists of two liquid solvents that are completely miscible. In detail, the solute is soluble in the first solvent, but insoluble in the second solvent, defined as the antisolvent. The solution, consisting of the solute and the first solvent, is allowed to drop into the antisolvent phase by inducing its supersaturation state; this leads to both solute precipitation and micro or sub-micro particle formation. Nanoparticle size and distribution depend on several parameters, such as the injection rate of the solvent into the antisolvent phase, the stirring speed, and the solvent/antisolvent ratio [18] (Figure 3). This process is currently the most-adopted preparation technique for the production of zein-based nanoparticles [55].

This method has the advantages of being cost-effective, providing high encapsulation efficiency [18], showing a good particle size distribution, and being both simple to implement and easily adaptable for industrial scale-up operations. Therefore, this methodology is widely applied to the production of protein-based nanoparticles [56]. The inherent difficulties in the optimal choice of solvent and antisolvent can lead to limitations and constraints in the mechanism of protein-based particle preparation, as the proteins may retain the same solubility as the incorporated and active compounds if the latter are bioactive substances [7,57].

**Figure 4:**
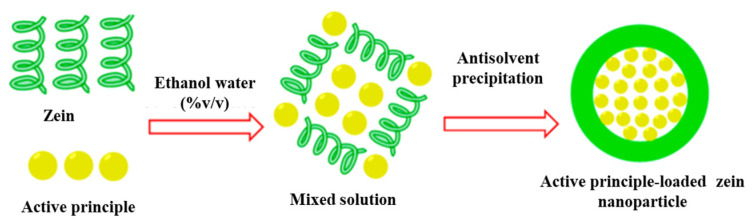
Schematic diagram of active compound encapsulation in zein particle [58].

Yan et al. demonstrated that the obtained size distribution of zein-based particles is influenced by the selected alcoholic compound, as it is affected by the evaporation rate of the solvent. Furthermore, the nanoparticle properties are strongly modified by other parameters, such as initial concentration, solvent/antisolvent ratio, pH, and stirring rate [26]. In the current literature, several studies have reported on the application of this technique in the production of nanocarriers for the encapsulation of active principles. Zein molecules and active substances, such as natural polyphenols, polypeptides, vitamins, minerals, and other nutraceuticals, are dissolved in an aqueous solution of ethanol at 60–90% (*v*/*v*); subsequently, the zein structure self-assembles by entrapping the bioactive compound. Encapsulation occurs due to the driving force of hydrophobic interaction and hydrogen bonding. However, process conditions could affect both encapsulation and the loading efficiency (Figure 4).

Typically, the loaded active compound is solubilized in the zein solution and then becomes part of the droplets falling into the antisolvent. This last step is a critical point in achieving encapsulation; however, the selection of the appropriate active compound to avoid solubilization in the antisolvent and optimize loading efficiency is equally important [58,59].

To prevent aggregation induced by the high surface hydrophobicity of zein nanoparticles in aqueous suspension, the effect of some surfactants (e.g., Pluronic F68, Polysorbate) can be exploited. In the case of Pluronic F68, the surfactant coats the protein nanoparticles, leading to an increase in the electrostatic repulsion or to the steric effects of the particles, thus reducing the hydrophobic attraction. In fact, Pluronic F68 surfactant features a stabilizing mechanism due to steric impedance; therefore, it affects the zein nanoparticles’ stability by imparting a negative surface charge as a result of the Zeta potential analysis instead of inducing the characteristic zein-positive surface charge [48,54,60]. Calliari et al. has loaded Hibiscus sabdariffa into zein nanoparticles without using surfactants as stabilizers, achieving an encapsulation efficiency of about 89%. Nevertheless, the zein nanoparticles exhibit a low stability in aqueous solutions with a significative reduction in encapsulation efficiency over a longer storage period (90–120 days) [61].

Table 1 reports the main results of the research activities related to the production of zein-based nanoparticles for use in different application fields, highlighting the main results related to the encapsulation efficiency (EE) of different active species.

### 3.2. Spray Drying

Spray drying is widely used for nanoparticle production due to its unique characteristic of tacking place in a single step. The process involves the atomization of a suspension or dispersion in small droplets, which are dried to solid particles using a continuous hot air flow. The resulting solid product is recovered through a series of cyclones and filters [71,72].

Spray drying is a rapid, reproducible, economical, and continuous process, with very efficient control of particle size distribution. One of the main limitations is the drying phase, during which a collision between the particles occurs, due to hydrogen and van der Waals interactions; moreover, the limited choice of polymers, the loss of volatile compounds, and the difficulty of encapsulating water-soluble compounds can be mentioned as further disadvantages for this technique [73,74]. This technique is affected by different factors interfering with particle size distribution, yield, and output product, such as the inlet and outlet stream temperature, the drying gas flow rate, the inlet flow rate, and the pressure [71,75,76].

The spray-drying process is widely used in the production of protein-based nanoparticles. Typically, a solution is prepared by dissolving a known concentration of zein in a hydroalcoholic solution with a percentage of ethanol ranging from 60 to 90% *v*/*v*. The solution is placed under magnetic stirring and a known concentration of the active ingredient is added at a suitable percentage with respect to the solute weight. The solution, or the suspension obtained by slowly adding water, is atomized during the spray-drying process. Campión et al. have demonstrated the suitability of zein-based nanoparticles to promote the bioavailability of quercetin. In detail, two systems were investigated: zein nanocarriers with and without wheat germ oil, characterized by similar size distribution, shape, zeta potential, and hydrophobicity. The wheat germ oil addition improved the preservation of lipids, avoiding their degradation and increasing the quercetin bioavailability [77]. Coelho et al. studied the effect of vitamin B12 encapsulated within zein nanocarriers to improve the bioavailability of the active compound, reporting an encapsulation efficiency of 91% in the case of microparticles with a size of 3 μm and spherical morphology. Furthermore, the slow release kinetics of Vitamin B12 were assessed within the first 11 h, as only 50% of the loaded content was found to be released [78]. According to different studies, zein carriers have been used for the encapsulation of β-carotene by using glycerol acting as stabilizer. Different carrier/active substance ratios have been used; considering the highest ratio, an encapsulation efficiency of about 64% has been calculated. The results highlight that zein, as a carrier, shows excellent properties for bioactive encapsulation and controlled release; moreover, an improvement in terms of the permeability and bioavailability of β-carotene was found [79].

Table 2 reports on the main research activity outputs and applications in different fields; these have been carried out using several active compounds. The table highlights the most important results related to specific properties such as particle size distribution and encapsulation efficiency (EE).

### 3.3. Supercritical Antisolvent Process

The supercritical fluid (SCF) methodology has been widely employed in the production of nanocarriers/nanoparticles. In the current literature, several research studies have concluded that the use of supercritical CO_2_ significantly reduces the use of organic solvents, allowing greater control over the morphology and size of the produced particles. SCF-assisted processes consist of a SC-CO_2_ supercritical solution and a dissolved solid compound, which are depressurized in a precipitation vessel. The size of the precipitated particles can be measured in micrometers or nanometers [83].

The supercritical assisted injection in a liquid antisolvent (SAILA) process leads to the production of nanoparticles in water suspensions by using supercritical CO_2_ [84,85]. The resulting particle size is affected by the micro-mixing efficiency of the two liquids, depending on their surface tension. However, at high CO_2_ molar fractions, the surface tension of the foamed liquid is close to zero; thus, micro-mixing improves with the antisolvent triggering smaller particle sizes. Other parameters can influence the particle size, such as the gas/liquid ratio (GLR), the saturator temperature, and the solvent/antisolvent ratio.

The main advantages of this technique are the control of the particle size distribution, enabling changes in the operating conditions and the reduced presence of solvents in the final product. Nevertheless, the relevant costs associated with the equipment and process implementation severely reduce and limit the use of this technique.

Recently, this technique has been used by Palazzo et al. [86] for the encapsulation of luteolin within zein-based particles. The authors were able to obtain microparticles with a mean diameter of 1.20 µm by appropriately modifying the process conditions in terms of the pressure, the temperature, and the carrier/active compound ratio. An encapsulation efficiency of 82% and an improved bioavailability of luteolin were obtained at high zein/luteolin ratios (e.g., 20/1).

A further process which gains its advantage from the supercritical CO_2_ (scCO_2_) antisolvent effect is the so-called supercritical antisolvent (SAS) method; this is considered a viable alternative to the traditional antisolvent precipitation method. The process involves the preparation of a solution consisting of the solute and the active ingredient, both dissolved into a solvent miscible with scCO_2_, which is later sprayed through a nozzle into a vessel containing scCO_2_. Upon contact with scCO2, the solvent content of the nebulized solution instantly diffuses, generating supersaturation conditions of the solute and thus the formation of particles. Due to the use of scCO_2_, a moderate temperature is set up during this process and the resulting product is solvent-free or it contains a negligible amount. The properties of the obtained nanoparticles are influenced by the fluid–dynamic interaction between phase equilibria and mass transport and by their influence on nucleation and growth phenomena [87]. The SAS process has largely been used for the production of zein-based micro- and nanocarriers with potential applications in the medical sector [50,88,89]. Recently, increasing interest has been shown in the development of zein-based protein carriers by SAS process within the field of food applications also. Rosa et al. have studied the feasibility of precipitating a vitamin complex containing riboflavin, α-tocopherol, and β-carotene in zein microparticles for controlled release systems that are applied to food products. They found that the process parameters, such as pressure, temperature, solution flow rate, and antisolvent, significantly affect both the particle size and the precipitate yield. Following an optimization procedure, they obtained spherical particles with a mean diameter in the order of 8–18 µm, depending on the co-precipitated vitamin, with a precipitation yield ranging from 410 to 820 g kg^−1^ [90].

### 3.4. Coacervation

**Figure 5:**
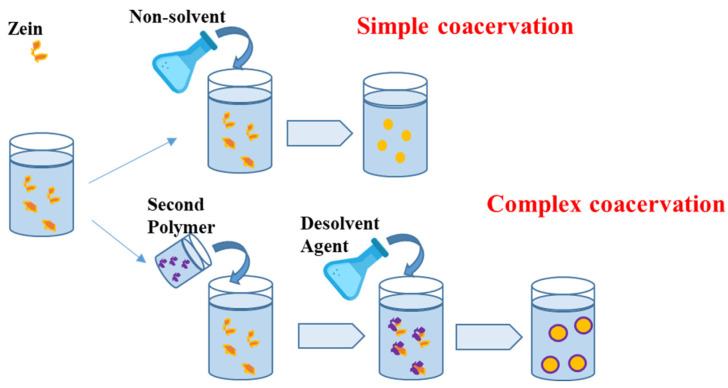
Schematic representation of the coacervation method.

Nanoparticle formation can be achieved by applying the coacervation technique, which is carried out under specific conditions in solutions or molecular dispersions that are held in a nonequilibrium thermodynamic state. Specifically, the separation takes place towards two immiscible and incompatible liquid phases by reducing the solubility of the solute, such as the polymer, in the solution/dispersion medium. The solubility of the solute is a function of the solvent polarity, the pH, the ionic strength, and the presence of electrolytes. The addition of a non-solvent agent leads to a change in the polymer structure, resulting in coacervation. (Figure 5).

The process of simple coacervation involves the addition of a substance that reduces the hydration to a hydrophilic colloidal solution, resulting in the formation of two phases: one rich in polymer (coacervate) and the other containing the supernatant [91]. In complex coacervation process, phase separation occurs due to the electrostatic interactions between two colloids, characterized by opposite charges [92].

The coacervation method is advantageous and relatively simple to implement, with low costs, a good level of scalability, a high encapsulation efficiency, and good reproducibility [93]. The control and optimization of the process variables allow for the obtained particle size to be tuned; however, there are some limitations in the effort to fully control the coagulation phase and the presence of cross-linker toxicity. Protein and polysaccharide coacervates have found several potential applications as carrier materials for essential oil encapsulation, among others. Simple coacervation was applied by Li et al. to attain zein nanocarriers loaded with limonene oil, when zein dissolution in water/propylene glycol (PG) led to particle formation but only under the appropriate operating conditions. As matter of fact, the net charge of the zein particle triggers an electrostatic barrier effect, which makes the encapsulation of limonene oil difficult; this can only be achieved at pH = 8 and with 65% *v*/*v* PG [94].

The complex coacervation technique has been successfully implemented to produce complex nanocarriers made of proteins and polysaccharides. Ma et al. have proposed a new nanocarrier model with a layered structure of zein and chitosan for the encapsulation of phenols and probiotics. It has been shown that the presence of the double structure increases the encapsulation efficiency, while improved encapsulation occurs at pH = 7 [95]. Farnad et al. studied the encapsulation of anthocyanins in zein, potato starch, and a mixture thereof by using both the simple and the complex coacervation methods. The reported results highlight a significant encapsulation efficiency higher than 80%, along with an antioxidant activity for pH ranging from 2 to 8 for the attained nanocarriers [96].

### 3.5. Emulsification Method

**Figure 6:**
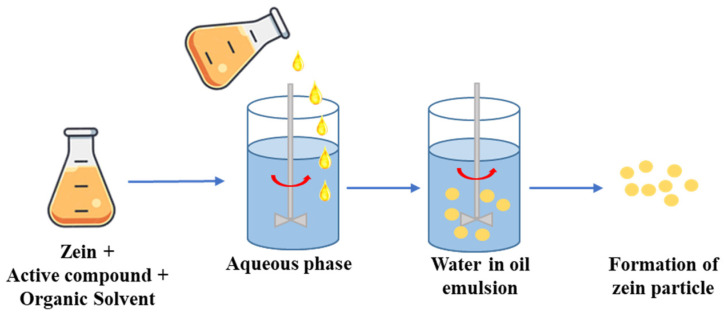
Schematic representation of emulsion process.

The emulsification method involves the dispersion of two immiscible fluids, comprising an oily and an aqueous phase. In this case, proteins and other molecules are dissolved or dispersed within the aqueous medium and then emulsified in a non-aqueous medium, such as oil, containing the active substance [97] (Figure 6).

The stability of the emulsion relies on various factors, including the particles size and their distribution, the density between the continuous and dispersed phases, and the chemical stability of the dissolved component [98]; therefore, the destabilization of the emulsion is attributed to a variety of processes, such as coalescence flocculation, sedimentation, creaming, Ostwad ripening, and phase inversion. To stabilize the emulsions, amphiphilic surfactant molecules are used to reduce particle surface tension. The main advantages of this method include the protection of the active ingredients from degradation processes, the improved absorption capacity and release rate of the active ingredient, and the improved ability to solubilize hydrophobic or oil-soluble compounds. However, emulsified particles are often unstable, which can result in short shelf life and phenomena such as creaming, cracking (breaking), flocculation, or phase inversion during the storage period [97].

To address these latter issues, Pickering emulsions have been introduced [99]; these involve the stabilization of the emulsion drops by means of one or more layers of particles. The presence of these coatings prevents coalescence among the droplets, at the same time delaying lipid oxidation. Although Pickering emulsions have a low absorption capacity and a low-fluidity structure, they offer greater chemical stability compared to emulsions stabilized with surfactants. Proteins, prepared with polysaccharide/protein complex or nanoparticles, can be used as emulsifiers and stabilizers in the Pickering emulsions due to their thickeners, gelling, and surface activity [100]; this enables the management of the physical localization and effectiveness of antioxidants as fundamental characteristics [101,102]. Zhao et al. observed the influence of protein-based nanoparticles on the interfacial distribution of antioxidants and on the oxidative stability of emulsions. Specifically, gallic acid (GA) was coated with zein particles by Pickering emulsions, demonstrating that the GA interfacial distribution is favored as the zein concentration increases. Consequently, coated GA shows a direct correlation among the zein concentration, the particle interfacial charge, and the oxidative stability [103]. Zhang et al. used zein nanoparticles and hyaluronic acid (HA) as a stabilizer to load bioactive compounds. The use of HA as a co-carrier improved the stability, increased the emulsion viscosity, reduced the lipid oxidation, and extended the shelf life of the nanoparticles. In particular, the implementation of a co-carrier for the encapsulation of astaxanthin has shown remarkable benefits such as antioxidant capacity, bio-accessibility, effective release, and greater storage stability [104]. Cui et al. proposed a combination of an anionic emulsifier and cationic zein nanoparticles to enhance emulsion stability for curcumin encapsulation. This combination inhibits the aggregation phenomenon, forming a thicker coating and protecting the active ingredient from chemical degradation [105].

### 3.6. Comparison of the Zein-Based Nanocarrier Properties Obtained by Different Methods

**Figure 7:**
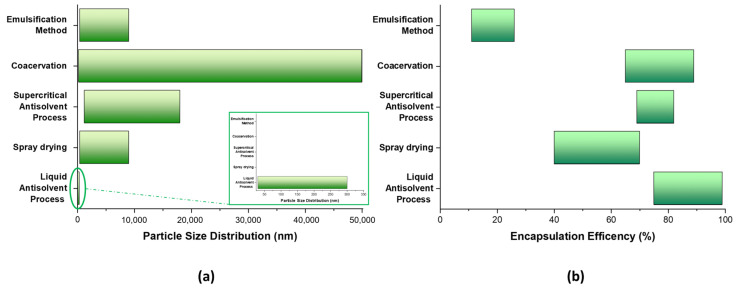
Range of values for PSD (**a**) and EE (**b**), based on the production technique specification.

In this section, the properties of zein-based nanoparticles, developed by several preparation methods, are discussed in terms of particle size distribution (PSD) and encapsulation efficiency (EE). The aim is to provide a comprehensive overview of these parameters in order to highlight the differences between the various preparation methods. Figure 7 shows a range of values for PSD (7a) and EE (7b); these are based on the production technique specification, as indicated by the considered studies.

It is noticeable that, depending on the specific methodology, the particle size distribution is different and highly variable, leading to the production of particles with sizes ranging from the nanoscale to the micron scale. For some techniques, the PSD is characterized by a wide range of factors, i.e., the coacervation method, so the manufacturing process could be a strictly limiting factor for composite fabrication.

Analyzing the encapsulation efficiency data, it could be remarked that, regardless of the implemented method (except for the emulsification method), the achievable level ranges between 70 and 80%.

## 4. Potential Application Fields of Zein Nanocarriers

The current section will provide a complete, but not exhaustive, review regarding the industrial applications of protein-based nanoparticles as potential nanocarriers of active compounds in more prominent application sectors: the agricultural, packaging, and cosmetics sectors.

### 4.1. Zein-Based Nanocarriers in Agriculture

Pesticides are commonly used to combat insect and weed damage in crops, but their use is not species-specific [106]. Thus, concerns about environmental threats associated with their exposure [106], their toxicity to other organisms, and their ability to contaminate soil, water, grass, and other vegetation are important to note [107,108], in addition to their tendency to induce root dieback in plants [109,110]. Recently, the application of nanotechnologies has been proposed to reduce the damage associated with the use of chemical substances, offering, at the same time, the prospect of increased productivity through the adoption more sustainable agricultural practices [110]. Although different nanoparticles have already been developed, the research trend is moving towards the development of biodegradable and biocompatible systems that can reduce the effects of the toxicity of metal-based products and enhance the green nanotechnological processes; this could lead to increased energy efficiency, and increase practices of implementing bio-compatible and non-toxic solvents and reliance on natural sources, including plants, algae, animals, and microorganisms [106,109,111,112]. Despite the fact that several studies have demonstrated the efficacy of botanical insecticides, which are considered safer for humans and the environment compared to conventional chemical products, their poor physicochemical stability, high volatility, low resistance to thermal decomposition [113], their inherent storage constraints, and their reduced water solubility still limit their adoption. To address the primary limitation of easy degradability, the use of nanoparticle-based systems for the encapsulation of the active principle arises as a promising and viable idea to improve the stability, water solubility, and bioavailability of the compounds [114].

**Figure 8:**
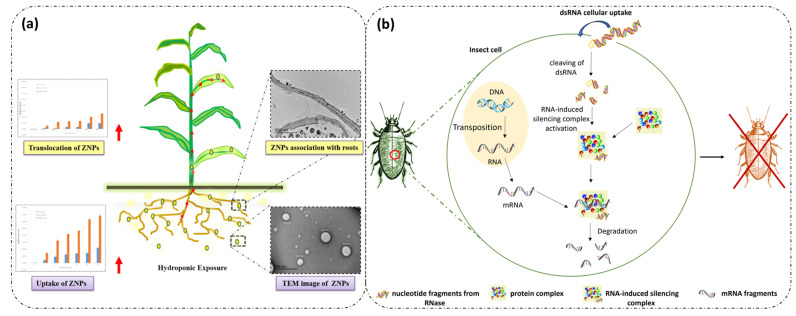
Representation of (**a**) uptake and translocation of positively charged zein nanoparticles [116] and (**b**) molecular mechanism of RNA-induced silencing complex.

Research efforts have promoted the use of zein nanoparticles due to their unique properties, making them a promising candidate for sustainable agricultural applications. Zein nanoparticles can be used to encapsulate various active compounds, including pesticides, fertilizers, herbicides, and plant growth regulators, protecting them from degradation and ensuring controlled release. Furthermore, zein nanoparticles are biodegradable, reducing the amount of chemicals needed, minimizing run-off, and preventing the potential contamination of soil and water sources. Prasad et al. investigated the uptake and translocation of positively charged zein nanoparticles (zein NPs) in sugarcane plants that were hydroponically exposed to NPs (Figure 8). The figure shows a schematic model of the uptake of zein-based NPs applied to the leaves of a plant by spraying; the silencing mechanism induced by the active ingredient, in this case RNA, during enzymatic degradation, is also shown.

The study examined the cationic interaction of zein NPs as a transport carrier for pesticide delivery and found that only a small fraction of it transfers from the roots to the leaves of the plant [115,116].

Kacsó et al. performed a degradation study of freeze-dried zein NPs, synthesized with different cationic and non-ionic surfactants, considering a limited soil pH range, namely from 4 to 9. It was found that nanoparticles degrade rapidly in alkaline systems; however, the nature of the surfactant can influence the kinetic profiles by reversing the trend [117]. Camara et al. investigated the behavior of zein-based NPs loaded into mixtures of botanical insecticides, which are released from the insect intestine upon enzymatic degradation. Their formulations showed an encapsulation efficiency of up to 95% with stability for 60 days. Enzymatic degradation was able to decompose the zein NPs and stimulate the active ingredient release, opening up a new strategy for the insect crop control through tailored and sustainable agricultural practices [118], providing a controlled release of “active” agents for pest control [113].

### 4.2. Zein-Based Nanocarriers in Food and Food Packaging

Nanotechnologies have been instrumental in the development of innovative solutions for food and food packaging, thanks to the unique properties of nanomaterials, enhancing color, morphology, texture, and food stability (shelf life and resistance to spoilage). By incorporating nanomaterials directly into food, more complex structures and functionalities can be achieved, such as increased antibacterial effects and longer food shelf life. The use of nanoparticles (NPs) based on carbon or organic polymers as preservatives in food packaging has raised concerns about their impact on human health, leading to increased safety control in the food industry. Despite limitations such as insolubility and non-uniform shape, size, and stability in storage, proteins are being explored as a means of producing nanoparticles and delivery systems for active molecules. Nanocomposites are a promising solution to overcome the limitations of traditional packaging by providing specific functionalities such as antimicrobial effects, degradation rate, thermal properties, barrier function, and food safety properties [7,119,120,121].

**Figure 9:**
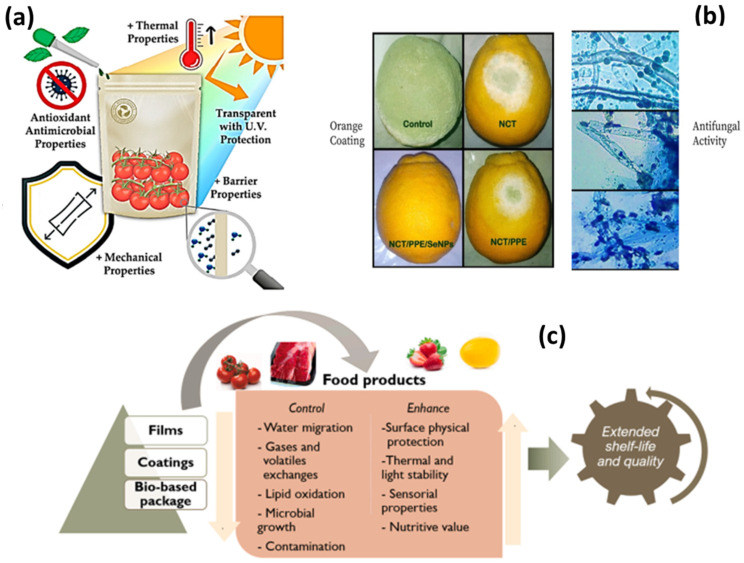
Schematic model: (**a**) advantages of bio-nanocomposites plastic packaging [119]; (**b**) antifungal application on citrus green of the protein-based NPs with pomegranate peels and nanochitosan as edible coatings for mold protection [120]; (**c**) effects of biodegradable and bioactive packaging on the food [123].

Protein-based nanocomposites have gained attention for their potential use in food packaging; this is particularly the case for zein, which has been deemed safe for packaging by the Food and Drug Administration (FDA). Zein has been used as a coating to create a sustainable packaging system that can prevent physical, chemical, microbial, sensorial, and nutritional changes in food products during handling and storage (Figure 9) [122,123].

In addition, zein can be used as a carrier for encapsulating hydrophobic/lipophilic food components such as lipids, fat-soluble vitamins, food dyes, flavorings, food supplements, nutrients, antimicrobials, and natural antioxidants.

Jiao et al. have investigated and developed an innovative method to improve the solubility, dispensability, stability, bioactivity, and oxidative degradation of lutein using zein as a carrier. The presence of zein as a carrier increased the bioavailability of lutein, with an encapsulation efficiency of more than 85% [124]. Zhang et al. investigated the mechanical interactions between zein nanoparticles and bovine serum albumin (BSA) as a potential application for the encapsulation of active compounds in the food industry. Their results showed that the optimal mass ratio is equal to 5:1, and the particles have excellent properties in terms of spherical shape and stability, which decrease with increasing BSA content. Ma et al. proposed a new zein–chitosan bilayer microencapsulation system and investigated the protective and delivery effects of polyphenols and probiotics. They observed an influence of pH, with increased interaction forces occurring as pH increases. The presence of a co-carrier increases the survival of probiotics compared to free probiotics under simulated digestion conditions and provides good stability during storage. Furthermore, it counteracts their loss during the simulated pasteurization process by blocking their thermal conduction [21,95,124,125].

### 4.3. Zein-Based Nanocarriers in Cosmetics

Due to their unique properties, nanoparticles have gained considerable interest in recent years as delivery systems for active molecules in dermatological applications. These systems offer several advantages, such as improved skin penetration and controlled release of active ingredients. The unique properties of nanoparticles offer significant benefits to consumers by enhancing the effectiveness of cosmetic products [126,127,128].

In particular, cosmetic substances require physicochemical properties to easily penetrate the epidermal layer. Only a few substances possess these characteristics, including low molecular weight, solubility in water and oil, and a low melting point. Various types of nanomaterials, such as organic and inorganic nanoparticles, nanocrystals, and polymeric nanoparticles, are widely used in cosmetics. Among these, liposomes, in particulate form, are the most-studied materials. The presence of a phospholipid bilayer affects their delivery properties, but their small size facilitates penetration through the epidermis. Despite these advantages, their limited chemical–physical stability and the difficulties that arise in large-scale production restrict their application [128,129].

**Figure 10:**
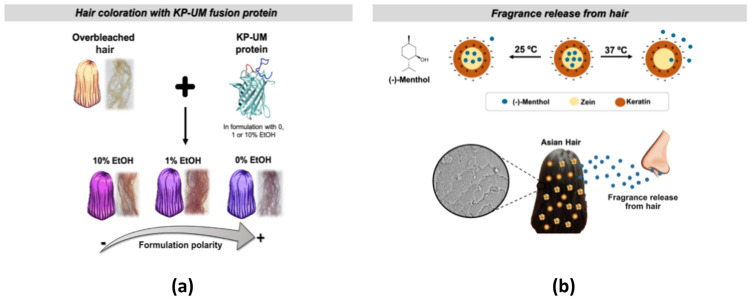
Effect of keratin (KP) and ultramarine (UM) protein-based NPs on hair coloration and scent (**a**); effect of temperature on fragrance release from keratin–zein particles deposited on the hair (**b**) [131].

Protein-based formulations have been demonstrated to have numerous benefits in cosmetics, such as improving hair properties and serving as particles for fragrance encapsulating and delivering. Due to their properties, proteins, protein hydrolysates, and peptides offer innovative solutions for new cosmetic products. They not only impart various properties to the hair, but also protect it from external agents and mitigate or prevent the negative effects of cosmetic compounds. Traditional carriers used for fragrance delivery in cosmetics have some limitations, such as low penetration, low stability, and controlled release of active ingredients [130,131]. Figure 10 shows the effect of protein-based nanoparticles on the release of color and encapsulated fragrance.

In particle preparation, zein has been used as a keratin co-carrier for the encapsulation of linalool and menthol fragrances. Tinoco et al. have shown that the presence of keratin increases zein particle stability, protects fragrances during the storage period, and shows an encapsulation efficiency of up to 76% [130]. To improve the release of fragrances, Ji et al. have tried to encapsulate cinnamaldehyde (CA) in a zein–chitosan (CS) composite nanocarrier, exploiting their antimicrobial activity since CA is not only a fragrance but also has antimicrobial activity. The interactions of CA with the zein–chitosan composite carrier significantly reduced its volatilization rate. The mass ratio of zein/CS led to different structures, influencing their encapsulation efficiency, release properties, and microbial activities. However, the presence of the co-carrier slowed down the release of CA. This system presents great potential for the cosmetics industry thanks to its controlled release properties and additional antibacterial activity [132].

### 4.4. Miscellaneous Applications

Thanks to their peculiarities, like availability, biodegradability, low-toxicity, and their different shape versatility (particles, membranes, films, and scaffolds), protein-based nanocomposites, particularly zein-based ones, are used in applications in fields or industries that are different to those previously discussed. For example, they can be used in the production of bio-based adhesives [133] or in the textiles industry to impart hydrophobicity and antimicrobial activity [134]. However, their most common applications are in the biomedical field and the pharmaceutical industry [87]; their use is in the pursuit of developing systems that can be used for targeted drug delivery, bioimaging, or soft tissue engineering. This is mainly because they correspond to the typical size of the natural functional units of living organisms, which allows a better interaction with biomolecules. Several studies have shown that zein NPs loaded with different drugs, both hydrophobic and hydrophilic, can be used as drug delivery nano systems, designed to localize drugs at target sites through site-specific supply as a potential effective therapy in the treatment of cancers and other diseases [135,136]. Recently, quantum dots (QDs) have been incorporated into zein nanoparticles, where strong hydrogen bonding and hydrophobic interactions have been driving forces in the production of nanocomposites for bioimaging and drug-delivery applications [137]. The use of zein-based electro-spun nanofibers is of a great interest in academic research due to their high resistance to microorganisms, flexibility, compatibility with the human body, biodegradability [138], and, in some cases, specific antimicrobial activity. However, the use of zein in the medical field and in the development of carriers for drug delivery has been widely discussed and presented in the literature and it is not the focus of this review, as previously stated.

## 5. Conclusions and Perspectives

Nanomaterials, particularly those based on proteins, have attracted considerable attention in several industrial sectors. The recent literature demonstrates a concentrated focus on employing proteins for the development of nanoparticles and nanocarriers with optimal efficiency in releasing suitable active compounds with specific targets and/or functions. This emerging field, which is well established in the context of smart materials that are able to respond and adapt to external stimuli, is significantly enhanced by the unique properties of protein-based nanomaterials, such as sustainability, biodegradability, and reproducibility. This review provides an overview of the current interest in zein-based nanomaterials, the different approaches for their production, and their potential industrial applications.

Zein possesses distinct characteristics, such as non-toxicity, biocompatibility, and biodegradability, as well as being a renewable and economical resource. Its highly hydrophobic nature allows for the formation of stable particles without requiring chemical bonds, making it an ideal candidate for the fabrication of nanocarriers capable of protecting and controlling the release of active compounds. Evidence from the literature shows that, in using different preparation methods, it is possible to produce complex zein particles as nanocarriers, incorporating hydrophilic and hydrophobic active compounds, with sizes ranging from a few tens of nanometers up to hundreds of nanometers, and with both narrow and broad size distributions.

Despite the various methods that are employed to enhance the encapsulation efficiency of zein-based nanoparticles, the main challenge remains in the chemical stability of these systems during storage. Zein nanoparticle suspensions are widely acknowledged for their poor colloidal stability, resulting in rapid agglomeration and precipitation. Notably, changes in pH influence their stability, with an increase in ionic strength leading to heightened van der Waals interactions and hydrophobic effects. This evidence is driving recent research toward the enhancement of chemical stability, such as in the dark or at 4 °C storage, by using co-carriers and/or surfactants as additives to improve the active substance dispersion by reducing NP aggregation in the final products.

A potential future development in the application of zein-based nanoparticles lies in the field of cultural heritage, which, as far as we know, is relatively unexplored. Research is currently focusing on the development of green solutions to reduce the potential harmful environmental and health effects of product applications for the cleaning, consolidation, and protection of artworks. Therefore, water-based products with adequate hydrophobicity and good stability under the conditions of environmental agents, organic solvent-free products, and highly transparent polymers derived from renewable sources have been proposed. However, these materials have not achieved satisfactory properties or performance in terms of stability, reproducibility, or durability. Meanwhile, the need to protect artefacts from weathering has driven research towards the development of nanostructured materials. In this scenario, the use of zein nanoparticles as carriers, for example, to encapsulate anti-corrosion and anti-fungal agents, is extremely interesting and could be a viable alternative to traditional products that have shown their limitations in terms of performance and durability in the conservation of cultural heritage.

## Figures and Tables

**Table 1:** Zein nanocarriers by liquid antisolvent process: properties and encapsulation highlights. PSD = particle size distribution.

Active Compound	Co-Carrier	Application	Properties and Encapsulation Highlights	Ref.
Geraniol + Eugenol Geraniol + Trans-Cinnamaldehyde	-	Agriculture	- PSD in the range of 234–282 nm; - Preservation of EO against rapid degradation; - Extension of active compound release overtime; - Low cytotoxicity; - Colloidal stability over 120 days; - EE up to 99%.	[62]
Limonene + Carvacrol	-	Agriculture	- PSD ~100 nm; - Enhanced antibacterial activity; - Good distribution in the midgut tissue of insects (in vivo tests); - EE up to 90%.	[60]
Neem oil	-	Agriculture	- PSD ~200 nm; - Colloidal stability at 120 days; - Not phytotoxic; - EE up to 80% until 90 days.	[48]
Quercetin	Carboxymethyl dextrin (CMD)	Food	- PSD in the range of 100–300 nm; - enhanced physical and chemical stability; - Improved antioxidant and antibacterial activities; - EE up to 80% with CMD coating.	[63]
Oregano Thyme	-	Food	- PSD in the range of 130–160 nm; - Colloidal stability up to 2 months when stored at 20 °C; - Enhanced antimicrobial activity against Gram^+^ bacteria; - Prevention of EO baking process degradation; - EE−79.6% for oregano - 93.2% for thyme.	[54]
Naringenin	Hyaluronic acid (HA)	Food	- PSD~210 nm; - NPs pH-stability in the range of 4.0 to 8.0; - Improved antioxidant capacity; - EE ~80% with HA addition.	[64]
Quercetin Resveratrol	carboxymethyl cellulose (CMC)	Food	- PSD ~220 nm; - CMC-coated particles intensify their electrostatic and steric repulsion, reducing their aggregation; - EE up to 25.1% for Resveratrol - Up to 3.5% for Quercetin.	[65]
Kaempferol α-Tocopherol	DNA DNA-quercetin complex	Food	- PSD in the range of 80–200 nm; - DNA coating reduces particles aggregation and improves storage stability; - EE 92.90% α-Tocopherol - 97.02% kaempferol.	[66]
Vitexin-rhamnoside	Pectin	Food	- PSD in the range of 230–250 nm; - Active compound slow release; - Increased bioavailability of active compound; - EE 67.6%.	[67]
Phlorizin (PHL)	Gum Arabic (GA)	Food	- PSD in the range of 120–165 nm; - GA co-carrier improves both PHL encapsulation and NPs thermal stability; - Enhanced water-holding capacity and yogurt antioxidant activity; - EE 44.68%.	[68]
Astilbin	Caseinate	Agriculture, Food	- PSD in the range of 132- 152.9 nm; - Colloidal suspension stabilization and prevention of the zein particles aggregation due to caseinate addition; - EE about 80%.	[69]
Thymol	-	Cosmetic, Food	- PSD in the range of 119.1–167.0 nm; - Improved thermal stability; - Enhanced antibacterial activity; - EE 64.98%.	[70]

**Table 2:** Zein nanocarriers by spray drying: properties and encapsulation highlights. PSD = particle size distribution.

Active Compound	Co-Carrier	Application	Properties and Encapsulation Highlights	Ref.
Green Jelly Leaves (GJL)	Hydroxypropylmethyl cellulose (HPMC)	Food	- PSD in the range of 320–1250 nm; - Reduced zein agglomeration; - Tailored release kinetic as a function of pH; - EE between 50 and 70%.	[80]
Eugenol	pectin, sodium caseinate (NaCas)	Food Agriculture	- PSD in the range of 400–500 nm; - PSD in the range of 186–400 nm depending on NaCas concentration; - Enhanced storage stability.	[81]
a-Tocopherol (TOC)	cyclodextrin (CD)	Food Agriculture	- PSD in the range of 1–9 µm; - Improved antioxidant activity; - Good dispersion and solubility; - EE of about 42%.	[82]
vitamin B12		Food	- PSD ~3 µm; - Improved bioavailability of the active compound; - Slow release kinetics; - EE ~91%.	[78]
β-carotene	Glycerol	Food	- PSD in the range of 499–2849 nm - Improved permeability and bioavailability; - Enhanced controlled release; - EE ~64% with highest zein/β-carotene ratio.	[79]

## Data Availability

The review contains consolidated data and no new data were created.
