# Correction: Oleandro et al. Zein-Based Nanoparticles as Active Platforms for Sustainable Applications: Recent Advances and Perspectives. *Nanomaterials* 2024, *14*, 414

**DOI:** 10.3390/nano15151179

**Published:** 2025-07-31

**Authors:** Emilia Oleandro, Mariamelia Stanzione, Giovanna Giuliana Buonocore, Marino Lavorgna

**Affiliations:** 1 Institute of Polymers, Composites and Biomaterials-CNR, Piazzale E. Fermi 1, 80055 Portici, Italy; emilia.oleandro@ipcb.cnr.it (E.O.); giovannagiuliana.buonocore@cnr.it (G.G.B.); marino.lavorgna@cnr.it (M.L.); 2 Institute of Polymers, Composites and Biomaterials-CNR, Via Previati 1/E, 23900 Lecco, Italy

In the original publication [1], “Verdolotti, L.; Lavorgna, M.; Oliviero, M.; Sorrentino, A.; Iozzino, V.; Buonocore, G.; Iannace, S. Functional Zein–Siloxane Bio-Hybrids. *ACS Sustain. Chem. Eng.* **2014**, *2*, 254–263” was not cited. The citation has now been inserted in the caption of Figure 2, within Section 2. Zein: Structure, Properties, and Current Applications. It reads as follows:

**Figure 2.** Three-dimensional structural representation of zein, adapted from [31].

Following this correction, the order of some references has been adjusted accordingly. The authors state that the scientific conclusions are unaffected. This correction was approved by the Academic Editor. The original publication has also been updated.
